# A Review of Recent Advances for the Detection of Biological, Chemical, and Physical Hazards in Foodstuffs Using Spectral Imaging Techniques

**DOI:** 10.3390/foods12112266

**Published:** 2023-06-05

**Authors:** Chuanqi Xie, Weidong Zhou

**Affiliations:** 1 State Key Laboratory for Managing Biotic and Chemical Threats to the Quality and Safety of Agro-Products, The Institute of Animal Husbandry and Veterinary Science, Zhejiang Academy of Agricultural Sciences, Hangzhou 310021, China; cqxie@zaas.ac.cn; 2 Institute of Digital Agriculture, Zhejiang Academy of Agricultural Sciences, Hangzhou 310021, China

**Keywords:** spectral imaging, foodstuff hazards, contamination, detection, models

## Abstract

Traditional methods for detecting foodstuff hazards are time-consuming, inefficient, and destructive. Spectral imaging techniques have been proven to overcome these disadvantages in detecting foodstuff hazards. Compared with traditional methods, spectral imaging could also increase the throughput and frequency of detection. This study reviewed the techniques used to detect biological, chemical, and physical hazards in foodstuffs including ultraviolet, visible and near-infrared (UV-Vis-NIR) spectroscopy, terahertz (THz) spectroscopy, hyperspectral imaging, and Raman spectroscopy. The advantages and disadvantages of these techniques were discussed and compared. The latest studies regarding machine learning algorithms for detecting foodstuff hazards were also summarized. It can be found that spectral imaging techniques are useful in the detection of foodstuff hazards. Thus, this review provides updated information regarding the spectral imaging techniques that can be used by food industries and as a foundation for further studies.

## 1. Introduction

Legal requirements stipulate that foods must meet specific safety standards [1]. However, issues regarding food safety persist. Food contamination is a common food safety issue that can occur during production, transportation, distribution, or storage, reducing the functional properties and nutritional values of foods and resulting in foodborne illnesses among consumers [2]. Generally, food contamination can be summarized as follows:

- Biological contamination: Bacteria and fungi are common biological contaminants in foods. For example, Bacillus cereus is a type of foodborne pathogen known to cause health problems, and thus, its detection is vital in foods [3,4]. Furthermore, the early detection and identification of aflatoxin are similarly critical to prevent its entry into food chains [5].
- Chemical contamination: Pesticide residues in agricultural products are the primary chemical contamination that could cause serious health problems. Moreover, food adulteration and fraud have caused public concern worldwide [6]. For example, benzoic acid and melamine are commonly added to wheat flour and milk powder, respectively. Imitation and fake food materials are usually introduced for economic purposes [7]. However, their health consequences can be lethal. Thus, the screening and identification of food authenticity are significant for consumers. Furthermore, harmful organic substances, such as 5-hydroxymethylfurfural (5-HMF) and acrylamide in heat-processed foods, are also common chemical contaminants.
- Physical contamination: Exogenous foreign substances (from glass pieces to wood chips, stones, and metal pieces) that are not intended to be food components commonly affect food safety [8]. Moreover, endogenous foreign bodies in foods (such as fish bones and nutshell fragments) are also hazardous to consumers. Thus, detecting foreign bodies is vital for assuring food safety [9].

Such issues are of growing concern, especially in label-free and non-branded foods, and drive the demand for fast and reliable methods to detect foodstuff hazards [10]. Traditional analytical and detection techniques including high-performance liquid chromatography (HPLC), mass spectrometry (MS), and enzyme-linked immunosorbent assays (ELISA) are not suitable for non-destructive detection in food industries [11]. In addition, these methods tend to be high-cost, labor-intensive, inefficient, and time-consuming [12]. Thus, rapid, accurate, and non-destructive food safety inspection demand has increased [13]. In terms of specificity, sensitivity, simplicity, low cost, rapidity, and non-destructivity, spectral imaging techniques are effective tools for evaluating food safety [6].

This review provides critical insight into the challenges and development trends in spectral imaging techniques for detecting foodstuff hazards. Detection methods using various spectral imaging techniques (ultraviolet, visible and near-infrared (UV-Vis-NIR) spectroscopy, terahertz (THz) spectroscopy, hyperspectral imaging, and Raman spectroscopy) are discussed from the three abovementioned perspectives (biological contamination, chemical contamination, and physical contamination). Machine learning algorithms were carried out to establish regression and classification models for detecting foodstuff hazards. The main objectives of this review were: (1) to discuss recent advances in various spectral imaging techniques in the detection of hazards in foods; (2) to compare the advantages and disadvantages of these techniques; and (3) to provide suggested detection techniques for specific hazards.

## 2. Spectral Imaging Techniques

Humans can only naturally observe objects within the visible spectral region (wavelengths of approximately 380–780 nm) [14]. The information in other spectral areas, such as near-infrared (NIR) and mid-infrared (MIR), can only be collected using sensors. Spectrum information in UV-Vis-NIR regions usually contains sensitive information regarding the vibration of molecular bonds. Thus, it can detect biological and chemical hazards in foods. At the same time, imaging information effectively detects physical hazards. Therefore, features of spectral imaging techniques should be considered according to the specific study and application requirements. The advantages and disadvantages of the most common spectral imaging techniques are listed in Table 1.

- UV-Vis-NIR spectroscopy has numerous advantages including easy operation, rapidity, non-destructive operation, in situ application, online application, low cost, and portability. Thus, it can be used to detect biological and chemical contamination. However, only spectrum information can be obtained using such a technique, not imaging information [15,16,17].
- THz spectroscopy covers the spectral region of 0.03–3 mm. It is used mainly to detect chemical and physical contamination because of its rapidity, reliability, non-destructivity, non-ionization, and spectral fingerprinting characteristics. However, the disadvantages of this technique include its high cost, the strong absorption of THz spectroscopy radiation due to water, scattering effects, limited penetration, limited sensitivity, and low limit of detection (LOD) [9,12].
- Hyperspectral imaging combines spectral and imaging features to detect biological, chemical, and physical contamination in foodstuffs. In addition, the spatial imaging features provide the visualization of objects, thus enhancing visual clarity in detection. However, image processing and data analysis are highly complicated in hyperspectral imaging, and the cost is higher than spectroscopy techniques. In food contamination detection, researchers prioritize spectroscopy techniques when imaging information is not required [8,16,20].
- Raman spectroscopy is used to detect biological, chemical, and physical contamination because of its high specificity, high sensitivity, simplicity, non-sensitivity to water, and evident Raman fingerprint of target attributes. However, this technique is usually limited to small sample volumes [6,16,19].

Proper spectral imaging techniques should be used according to their advantages and disadvantages to obtain satisfactory detection results. The appropriate applications of spectral imaging-based methods for detecting foodstuff hazards are listed in Table 2 including the detection of biological, chemical, and physical contamination using UV-Vis-NIR spectroscopy, THz spectroscopy, hyperspectral imaging, and Raman spectroscopy. As is evident in Table 2, spectral imaging-based techniques combined with chemometrics have been shown to detect hazards in foodstuffs effectively.

## 3. Applications

### 3.1. UV-Vis-NIR Spectroscopy

**Figure 1:**
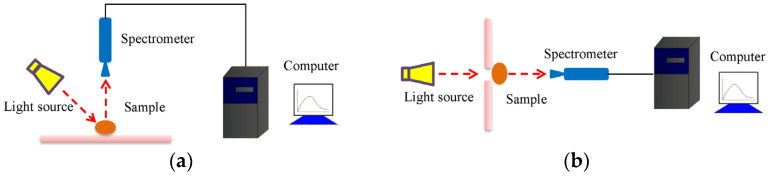
The schematic diagram of (**a**) the reflectance spectrum and (**b**) the transmittance spectrum.

UV-Vis-NIR spectroscopy covers the spectral wavelengths of 10–2500 nm (UV: 10–380 nm, Vis: 380–780 nm, and NIR: 780–2500 nm). Generally, UV-Vis-NIR spectroscopy is used to detect biological and chemical hazards in foods. The schematic diagram of the reflectance spectrum and the transmittance spectrum can be seen in Figure 1a,b.

#### 3.1.1. Biological Contamination

The contamination of foodstuffs by biological hazards, such as bacteria and fungi, can happen at any time between production and consumption. Spectral reflectance has been investigated for the detection of biological contamination in foods. For example, Tao et al. [23] used visible and near-infrared (Vis-NIR) spectroscopy to detect the surface contamination of peanut kernels with aflatoxin B1. Partial least squares-discriminant analysis (PLS-DA) based on total spectral absorbance was carried out to detect different contamination levels in the peanut kernels. The best detection accuracies were 88.57% and 92.86%, with 20 and 100 ppb thresholds, respectively. In addition, the random frog was investigated to identify effective wavelengths. PLS-DA models were built again, obtaining overall accuracies of 90% and 94.29%, with 20 and 100 ppb as the classification thresholds, respectively. These improved detection accuracies indicated that selected wavelengths perform better than full wavelengths. This study demonstrated that Vis-NIR spectroscopy could effectively classify peanut kernels contaminated by aflatoxin B1, thus, had the potential for use in large-scale online detection and non-destructive screening. In another study, Cheng et al. [21] used UV-Vis-NIR spectroscopy to classify corn kernels contaminated by different aflatoxin levels. The random forest (RF) classification model was built to identify contaminated samples, achieving overall accuracies of 95.3% and 94.8% in the training and testing sets, respectively. Moreover, the wavelengths 390, 540, and 1050 nm were found to play vital roles in the classification. This study, thus, concluded that UV-Vis-NIR spectroscopy could be used in the classification of single corn kernels contaminated by aflatoxin. Fourier transform near-infrared (FT-NIR) spectroscopy was also used to detect aflatoxin B1 in corn [24]. After pre-processing by SNV, ant colony optimization (ACO) and NSGA-II algorithms were investigated to select characteristic wavelengths. Based on the selected wavelengths, the NSGA-II-back propagation neural network (NSGA-II-BPNN) obtained the best result with a correlation coefficient of 0.9951 in prediction. This study showed that the NSGA-II algorithm could obtain effective wavelengths for predicting aflatoxin B1 in corn.

In addition to aflatoxin, the detection of toxigenic fungi in wheat kernels via Vis-NIR spectroscopy has also been reported. Shen et al. [22] applied principal component analysis (PCA) and linear discriminant analysis (LDA) to identify infected samples, and the LDA obtained accurate classification rates of 75–100% for different fungal strains (Fusarium and Aspergillus) as well as 88.3–100% for different infection levels. In addition, the partial least squares (PLS) model was carried out to predict colony counts in wheat kernels, with a coefficient of determination in prediction (R_p_^2^) of 0.89 and a residual predictive deviation (RPD) of 3.03, indicating the excellent performance of the prediction model [59]. Thus, this work proved Vis-NIR to be an effective tool in the early detection of toxigenic fungi contamination in wheat grains and introduced a novel approach for reducing the risk of mycotoxin entry into food chains.

#### 3.1.2. Chemical Contamination

Because of the molecular vibration of chemical groups such as C-H, N-H, and O-H, spectroscopy, especially for NIR, has been widely used in chemical contamination in foodstuffs. Pesticide residues and their metabolites in foods commonly cause adverse effects on humans [27]. Thus, Vis-NIR spectral reflectance was carried out to detect pesticide residues (chlorothalonil, imidacloprid, and pyraclostrobin) on the surface of Hami melon [25]. In this study, a one-dimensional convolutional neural network (1D CNN) was used to identify the samples contaminated by the pesticide residues, obtaining classification accuracies of 95.83% for four-class samples (control, chlorothalonil, imidacloprid, and pyraclostrobin) and 99.17% for two-class samples (with and without pesticide residues). The results based on 1D CNN outperformed those obtained by CNN, PLS-DA, and support vector machine (SVM) models. Moreover, Sankom et al. [28] used FT-NIR combined with Fourier transform mid-infrared (FT-MIR) spectroscopy to detect profenofos residues in Chinese kale, cabbage, and chili spur pepper. The original spectral absorbance data were pre-processed via 1st derivative and SNV. The PLS models based on FT-NIR subsequently obtained the best prediction results with R^2^ of 0.97 for Chinese kale, 0.88 for cabbage, and 0.96 for the chili spur pepper, thus showing the potential of FT-NIR spectroscopy as a promising screening tool for the detection of pesticide residues in vegetables. NIR spectroscopy was also carried out to detect boscalid and pyraclostrobin in strawberries [27]. After pre-processing (1st and 2nd derivative, multiplicative scatter correction (MSC), and standard normal variate (SNV)), the spectral absorbance data were used to build PLS models, subsequently obtaining correlation coefficients in the prediction of 0.93 for boscalid and 0.83 for pyraclostrobin. In another study, Rodriguez et al. [26] used NIR spectroscopy to detect chlorpyrifos-methyl pesticide residues in rough, brown, and milled rice. They built PLS models based on the spectral absorbance data pre-processed by mean centering, SNV, MSC, and derivatives. Though only one type of pesticide (chlorpyrifos-methyl) was identified, the results were promising, with R^2^ of 0.702–0.839 for rough rice, 0.722–0.800 for brown rice, and 0.693–0.789 for rough rice obtained. Furthermore, qualitative detection effectively achieved classifications of 77.8–92.6% for rough rice, 79.6–88.9% for brown rice, and 94.4–100% for milled rice. The chlorpyrifos on bok choi was also detected using NIR spectroscopy [31]. The researchers used different machine learning algorithms (PLS-DA, SVM, artificial neural network (ANN), and principal component-artificial neural network (PC-ANN)) to identify chlorpyrifos on bok choi. Finally, the accuracy, precision, recall, and F1-scores were 1.0 for PLS-DA, SVM, and PC-ANN algorithms on the unknown dataset. The results demonstrated that NIR spectroscopy combined with machine learning effectively detects pesticide residue. All of these findings proved the effectiveness of NIR spectroscopy in detecting pesticide residues in foods.

Besides pesticide residues, the harmful organic substances in heat-processed foods are also hazardous to humans. For example, 5-HMF is carcinogenic to humans. Thus, Apriceno et al. [29] detected 5-HMF content in honey using NIR spectroscopy and obtained an R_p_^2^ of 0.98. The excellent result indicated that the NIR spectroscopy technique has the potential to can act as an early warning against the critical 5-HMF threshold in food industries. However, this study only investigated 41 samples, which might affect the stability of the models. Acrylamide, a neurotoxin with carcinogenic properties, is another common harmful organic substance in fried and baked foods. Smeesters et al. [30] classified different levels of acrylamide in potatoes using Vis-NIR spectroscopy and obtained the classification of 92% by LDA. These studies provided a reference for the large-scale detection of harmful organic substances in food processing industries.

### 3.2. THz Spectroscopy

**Figure 2:**
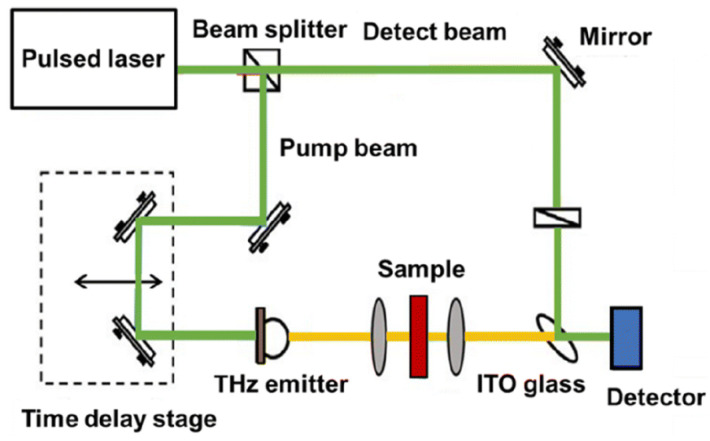
The schematic diagram of the THz spectroscopic imaging system [9].

THz spectroscopy covers the spectral wavelengths of 0.03–3 mm (frequency of 0.1–10 THz), which locates in another spectral region different from Vis-NIR. The vibration and rotational energy levels of most biological molecules, such as DNA, protein, and amino acids, are located in this spectral range [60], making THz spectroscopy useful in detecting toxic and harmful compounds, antibiotics, and foreign bodies in foods [12]. The schematic diagram of the THz spectroscopic imaging system can be seen in Figure 2. This review listed some typical studies regarding food contamination (chemical and physical) detection using THz spectroscopy.

#### 3.2.1. Chemical Contamination

Benzoic acid is sometimes used to preserve certain foods. However, long-term consumption of this chemical additive can cause cumulative poisoning in the liver [33]. Thus, various studies have used THz spectroscopy to detect benzoic acid in wheat flour. Generalized regression neural network (GRNN) and back propagation neural network (BPNN) models were built based on orthogonal PCA scores transformed from THz spectra, and the GRNN model obtained the best detection result with a correlation coefficient of 0.85 in the prediction [33]. Melamine can increase the protein content in foods and is, thus, commonly used as an illegal additive in milk powder [18], making detecting melamine highly significant for food safety. Therefore, Sun et al. [34] detected melamine in milk powder using THz spectroscopy, building a multiple linear regression (MLR) model based on a pair of variables at 2.04 and 2.34 THz, as suggested by correlation analysis. The researchers then compared MLR with the PLS model based on full spectral wavelengths. They found that the MLR model performed excellently, obtaining a correlation coefficient of 0.97 and demonstrating that THz spectroscopy helps detect melamine in milk powder.

Furthermore, THz spectroscopy is also effective in detecting pesticide residues in foods. For example, Qin et al. [32] applied THz time-domain spectroscopy to detect pesticide residue (carbendazim mixtures) in packaged foods. This study investigated different weight ratios of carbendazim in polyethylene and rice powder, and pure carbendazim, polyethylene, and rice powder were subsequently effectively classified. It was shown that SVM performed excellently in the qualitative detection of carbendazim when the weight ratio was low. In addition, when PLS and support vector regression (SVR) were performed to detect carbendazim mixtures, the best result was obtained by SVR with the R of 0.9978. These findings proved that THz time-domain spectroscopy also effectively detects pesticide residue in packaged foods. Using THz spectroscopy, Qu et al. [39] detected pesticide residues (2,4-dichloro phenoxy acetic acid) in Zizania latifolia, rice, and maize. Four baseline correction methods (asymmetric least squares (AsLS) smoothing, adaptive iteratively reweighted penalized least squares (airPLS), background correction (Backcor), and baseline estimation and denoising with sparsity (BEADS)) were carried out to eliminate spectral baselines. The results showed that the detection limit and accuracy of 2,4-dichloro phenoxy acetic acid residues were improved by baseline correction. In another study, THz spectroscopy was carried out to detect three pesticides (6-benzyl amino purine, 2,6-dichloro benzonitrile, and imidacloprid) in wheat flour [35]. BPNN with parameter optimization (genetic algorithm (GA)) and wavelength selection (particle swarm optimization (PSO)) obtained the best prediction results with correlation coefficients in the prediction of 0.9913, 0.9948, and 0.9923 for 6-benzyl amino purine, 2,6-dichloro benzonitrile, and imidacloprid, respectively. This study demonstrated the feasibility of THz spectroscopy in detecting pesticides with low concentrations.

#### 3.2.2. Physical Contamination

Due to its non-destructivity, non-ionization, and spectral fingerprinting characteristics advantages, THz spectroscopic imaging was also used to detect endogenous contamination in a complex food matrix [9]. The endogenous foreign bodies in walnuts were first detected according to kernels and shells’ typical absorption spectrum features, and the classification accuracy of PCA achieved higher than 95%. The results showed that THz spectroscopic imaging could effectively identify shell contamination among walnut kernels. Shin et al. [38] analyzed the optical features (refractive index and absorption coefficient) of food materials (sugar and milk powder) and foreign substances (insects) in the THz frequency range. The absorption coefficients increased as the frequency increased, and the refractive indices decreased. Finally, foreign substances were identified based on the optical features of the food matrix and insects. Jiang et al. [37] detected foreign bodies (a stone, a metal screw, a glass fragment, and a wood chip) in wheat grain using THz reflection imaging. Image pre-processing (linear low-pass filtering) was carried out to improve the visual contrast between the foreign bodies and the grain samples. Ultimately, foreign bodies in the grain were detected and identified, thereby confirming the value of the THz reflection imaging technique. In another study, Wang et al. [36] detected foreign materials (aluminum shards) in sausages using THz spectroscopic imaging. The locations of contamination in the sausages were identified based on typical spectra, PCA, and discriminant analysis (DA), among which the correct classification rates of DA were up to 98.3–100%. This study provided a new technique for detecting foreign materials in foods.

Moreover, Ok et al. [40] inspected food quality (in chocolate bars, dried laver, red ginseng, and walnuts) using a large-scan-area sub-THz imaging system, in which foreign bodies (metal washers, rubber bands, pepper seeds, and polystyrene pieces) were well discriminated. The foreign bodies in fish were also detected using THz imaging and spectroscopy [41]. Both endogenous foreign bodies (fish bones) and exogenous foreign substances (metal, plastic, and wooden toothpicks) in fish were investigated in this study. Competitive adaptive reweighting sampling (CARS), uninformative variable elimination (UVE), and successive projections algorithm (SPA) were carried out to identify valuable features. Then, PLS-DA, LDA, and SVM were established to detect foreign bodies in fish. Finally, the best detection result was obtained by CARS-SVM with an accuracy rate of 99.56%.

### 3.3. Hyperspectral Imaging

**Figure 3:**
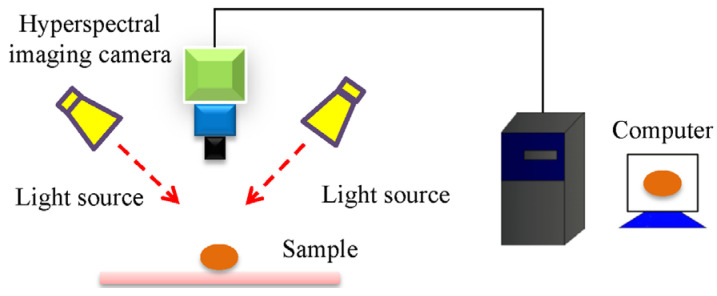
The schematic diagram of the hyperspectral imaging system.

Hyperspectral imaging is more powerful than spectroscopy techniques since it can also provide spatial information. Unlike spectral sensing, it can simultaneously provide spectral (from Vis to NIR) and imaging information (covering hundreds or thousands of wavebands). Thus, each pixel in the hyperspectral image has continuous spectrum information. However, hyperspectral imaging is more complicated than spectroscopy, and its parameters (such as moving speed, exposure time, and the vertical distance between the camera and samples) must be set before image collection. After that, the raw images must be calibrated using white and dark reference images based on Equation (1). The schematic diagram of the hyperspectral imaging system can be seen in Figure 3.

(1) $$I_{calibrated}=\frac{I_{r\mathrm{aw}}-I_{dark}}{I_{white}-I_{dark}}$$

#### 3.3.1. Biological Contamination

Due to the advantages of hyperspectral imaging, it performs well in detecting biological contamination in foods. For example, using hyperspectral imaging, Chakraborty et al. [43] detected aflatoxin B1 in maize kernels, a common mycotoxin and human carcinogen. PLS models were built to predict aflatoxin B1 concentrations in maize kernels naturally contaminated by Aspergillus flavus. After data pre-processing (via MSC and SNV), two classification models (PLS-DA and K-nearest neighbor (KNN)) were developed to classify the samples contaminated by different concentrations of aflatoxin B1. The PLS model acquired a good prediction result with the R_cv_^2^ of 0.82, while the KNN model, based on raw data, obtained the best classification accuracy of 98.2%. In the PCA score plot, the samples with low contamination levels (25, 40, and 70 ppb) were far from highly contaminated samples (200, 300, and 500 ppb).

Furthermore, the pixel-wise classification of the maize kernels developed via KNN and the aflatoxin B1 concentration distribution image based on PLS were close to the results obtained via HPLC. Overall, these results demonstrated that hyperspectral imaging could play an essential role in classifying maize kernels contaminated by aflatoxin B1 and predicting aflatoxin B1 concentration. In another study, hyperspectral imaging was used to detect and classify different levels (5, 10, 20, 30, and 50 ppb) of aflatoxin B1 contamination in pistachio kernels [42]. PCA showed that the control samples were far from all contaminated samples, and overall classification accuracies of 92.5% calibration and 91% validation were obtained via LDA. Moreover, stepwise multiple linear regression (SMLR) prediction models were used to obtain correlation coefficients in calibration and validation higher than 0.91 and, additionally, five wavelengths (708, 771, 892, 915, and 941 nm) were identified via PCA-loading, which also played vital roles in the aflatoxin B1 classification and prediction. The results of this study, thus, demonstrated that hyperspectral imaging is effective in the preliminary screening of pistachio kernels. A one-dimensional modified temporal convolutional network (1D modified TCN) based on hyperspectral imaging was also used to detect aflatoxin B1 in peanut kernels [44]. Four models (1D modified TCN, one-dimensional temporal convolutional network (1D TCN), one-dimensional long short-term memory (1D LSTM), and 1D CNN) were investigated, and 1D modified TCN achieved the best accuracies with 99.60% in training and 99.26% in testing.

In addition to aflatoxin, deoxynivalenol has also caused growing concern because of its prevalence in wheat. Thus, Femenias et al. [11] carried out hyperspectral imaging to detect and classify wheat kernel contamination by deoxynivalenol. The PLS models built using spectral reflectance information obtained an R_cv_^2^ of 0.72 in full cross-validation and an R_p_^2^ of 0.27 in independent validation. Moreover, the correct classification accuracy in the validation set of 62.7% was obtained via LDA for two categories of the samples. This work, thus, demonstrated the power of hyperspectral imaging in deoxynivalenol screening.

#### 3.3.2. Chemical Contamination

Besides biological contamination, this technique has also been used to detect chemical contamination in foods. For example, pesticide residues such as fenvalerate and dimethoate in lettuce leaves were detected by hyperspectral imaging [45]. After pre-processing via SNV, CARS, random forest-recursive feature elimination (RF-RFE), and SPA were carried out to identify valuable wavebands, and least-squares support-vector regression (LS-SVR) was subsequently built to predict the fenvalerate and dimethoate content. The CARS-SPA-LS-SVR achieved an R_p_^2^ of 0.8890 for fenvalerate, while the RF-RFE-SPA-LS-SVR achieved an R_p_^2^ of 0.9386 for dimethoate. In another study, the pesticide residues (lambda (λ)-cyhalothrin, trichlorfon, and phoxim) on garlic chive leaves were detected using shortwave infrared hyperspectral imaging, with pure water used as the control [47]. The signal-to-noise ratio (SNR) was improved by modified mean filtering (MMF), and outliers were eliminated using the isolated forest algorithm. The researchers subsequently explored the effectivity of different classification models (1D CNN, KNN, LDA, Naive Bayes (NB), RF, and SVM). They found that 1D CNN obtained the best classification results, with 98.5% in training and 97.9% in testing, thus providing a reliable tool for detecting pesticide residues on garlic chive leaves. In another study, Bai et al. [46] compared the performance of hyperspectral imaging with that of the spectroscopy technique. They detected sulfite dioxide residue on the surface of fresh-cut potato slices using hyperspectral imaging and NIR spectroscopy. The results showed that SVM based on whole hyperspectral imaging provided classification accuracies of 98.75% in calibration and 95% in prediction. After that, the Savitzky–Golay algorithm was investigated to identify effective wavelengths, achieving corresponding classification accuracies of 99.38% in calibration and 92.50% in prediction. Selected wavelengths tend to provide slightly lower classification accuracy in prediction than full wavelengths. However, the model based on selected wavelengths was simpler and faster, and the hyperspectral imaging produced better classification results than the NIR spectroscopy. It proved hyperspectral imaging also effectively detects sulfite dioxide residue on fresh-cut potato slices.

Veterinary drug residues are another common chemical contaminant in foods. Jiang et al. [48] detected veterinary drug residues (metronidazole, ofloxacin, salbutamol, and dexamethasone) in beef using hyperspectral imaging. CARS, PCA, and discrete wavelet transform (DWT) were investigated to reduce the dimensions of the hyperspectral imaging data. After that, CNN, a multilayer perceptron (MLP), SVM, and RF were built to identify the veterinary drug residues, and overall accuracies of 91.6%, 88.6%, 87.6%, and 86.2%, respectively, were obtained based on DWT. This study, thus, demonstrated that hyperspectral imaging is also effective in the detection of veterinary drug residues in foods.

#### 3.3.3. Physical Contamination

All the above studies focused on biological and chemical contamination in foodstuffs using hyperspectral imaging. Physical contamination in foods is also recognized as a common food safety issue. Researchers have, for example, investigated hyperspectral imaging to identify physical hazards in foodstuffs. Using hyperspectral imaging, Lim et al. [8] detected bone fragments embedded in chicken meat. Five bone fragments with lengths of approximately 20–30 mm and deboned chicken breast pieces with thicknesses of 3, 6, and 9 mm were investigated in this study. Spectral reflectance covering 987–1701 nm of the bone fragments embedded in the chicken meat was extracted. After that, PCA was carried out to visualize the spectral reflectance and, thereby, describe the varieties of samples. Finally, the chicken breast meat, bone fragments, and embedded bone fragments were well classified for samples of 3 mm thickness. However, the embedded bone fragments and chicken breast meat of 9 mm thickness could not be effectively classified.

The subtraction image algorithm (image 1153.8 nm–image 1480.2 nm) was then used to detect bone fragments in the chicken meat, and an accuracy of 93.3% was obtained. These findings indicated that hyperspectral imaging could also effectively detect foreign substances embedded in chicken meat. Endogenous foreign bodies, such as nutshell fragments, are also consumer safety hazards. Thus, the effectivity of hyperspectral imaging for identifying Chinese hickory nutshell fragments was investigated by Feng et al. [51]. The researchers compared a two-dimensional convolutional neural network and long short-term memory (2D CNN-LSTM), PCA-KNN, and SVM models. Lower classification results obtained by PCA-KNN and SVM indicated that food safety issues caused by the shell fragments would increase. However, the 2D CNN-LSTM model provided the best overall classification accuracy, at 99%. Moreover, visualization images of the foreign bodies (shell fragments) were produced, which were helpful for detection.

Exogenous foreign objects, such as insects, shrimp shells, thread, feathers, and plastic, have also been detected in seaweed via hyperspectral imaging. Kwak et al. [49] overcame the hyperspectral imaging limitation of low-speed inspection by incorporating dimensionality reduction and simplified operations. Seaweed and conveyor belts were first classified using the subtraction method, whereafter the standardization inspection was investigated to improve results. The proposed algorithm and SVM achieved 95% and 79% detection accuracies, respectively. Foreign materials (polymer, wood, and metal) in broiler breast meat were detected using hyperspectral imaging by Chung et al. [50], in which thirty different types of foreign materials of two different sizes (5 × 5 mm^2^ and 2 × 2 mm^2^) were identified. A fusion model, which combined Vis-NIR and shortwave infrared hyperspectral imaging, was used to obtain classification accuracies of 95%, 95%, and 81% for polymer, wood, and metal, respectively, all with the size of 2 × 2 mm^2^. The corresponding results were 100%, 100%, and 100% for polymer, wood, and metal, respectively, when the sample size was 5 × 5 mm^2^. This study also showed that the fusion model performed better than the individual Vis-NIR and shortwave infrared-based models by 18% and 5%, respectively. In another study, the foreign materials (polylactic acid, polypropylene, polyethylene terephthalate, and polyvinyl chloride) in soy protein meat were detected using hyperspectral imaging [52]. The hyperspectral transmission images were investigated, and the model (MSC-PCA-SPA-SVM) obtained the best classification accuracy of 95.00% in the validation. From this study, the localization of the analogous density foreign matter can be seen clearly from visualization images.

**Figure 4:**
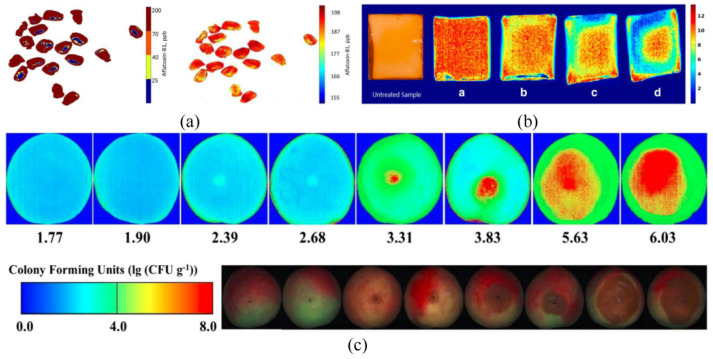
(**a**) Pixel-wise classification of maize kernels based on KNN and concentration distribution of aflatoxin B1 in maize kernels based on PLS [43], (**b**) distribution of surviving Listeria monocytogenes loads in dried eggs (a: 10 s; b: 15 s; c: 25 s; d: 30 s) [61], and (**c**) visualization of total colony counts in peach fruit [20].

Another advantage of hyperspectral imaging is that it can provide visualization images based on spatial information, thereby clearly detecting the distribution of foodstuff hazards. For example, the visualization images showing the distribution of aflatoxin B1 in maize kernels [43], surviving Listeria monocytogenes loads in dried eggs [61], and total colony counts in peach fruit [20] can be seen in Figure 4a–c. The principle underlying visualization images can be described as follows: Based on the spectral reflectance from hyperspectral imaging, a prediction model is built. The prediction model can be described as Y = f(x), where x is the spectral reflectance value, and Y is the chemical value. Each pixel in the hyperspectral image has a spectral reflectance value; thus, each pixel corresponds to one chemical value according to the equation. The chemical values of all pixels are then marked using various colors ranging from blue to red (from low to high). Finally, a visualization image showing the chemical values (such as the content of hazards in foodstuffs) is generated, significantly clarifying the detection process.

Hyperspectral imaging was also combined with other techniques such as electronic nose in food contamination detection. For example, Liu et al. [62] detected microbial content, total soluble solids, and titratable acidity in strawberries during decay. The results showed that the color, total soluble solids, and titratable acidity of infected strawberries were highly correlated with their microbial content. The SVM models based on principal components (PCs) performed better than the single dataset of hyperspectral imaging or electronic nose. The best results (R_p_^2^) were 0.925 for colony counts, 0.824 for total soluble solids, and 0.598 for titratable acidity. Furthermore, the visualization of microbial content distribution was generated using the SNV-SVM model. This study demonstrated the performance of the combined techniques for evaluating strawberry safety.

### 3.4. Raman Spectroscopy

**Figure 5:**
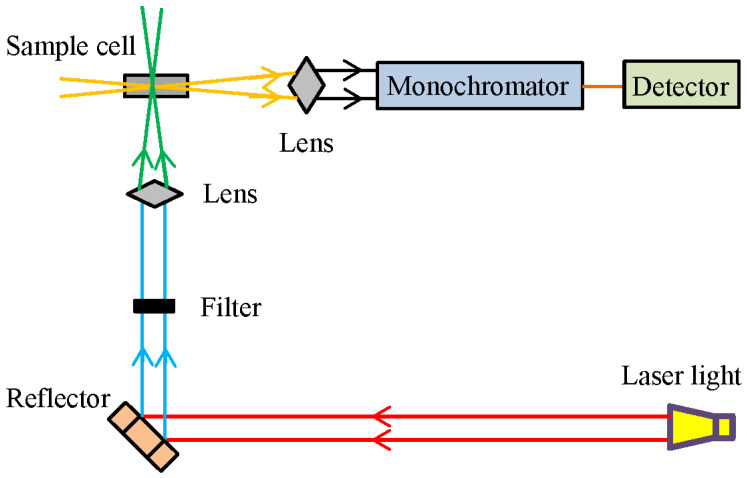
The schematic diagram of the Raman spectrometer [63].

Raman spectroscopy has been shown to effectively obtain information about molecular structures and the composition of samples [7]. Compared with other techniques, Raman spectroscopy is not affected by water within the samples and, thus, shows excellent performance in liquids [19]. Moreover, this technique can be utilized in microanalysis when combined with a microscopy system [58]. The schematic diagram of the Raman spectrometer can be seen in Figure 5.

#### 3.4.1. Biological Contamination

Raman spectroscopy was applied to detect foodborne pathogens (Escherichia, Listeria, Vibrio, Shigella, and Salmonella) by Vakilian [53]. The GA and PSO were used to optimize the architecture of ANNs for detecting the type of foodborne pathogens. Finally, GA-ANN and PSO-ANN obtained average accuracies of 0.89 and 0.93, respectively. Based on the Raman spectra of the cells, ATCC 14028 (the strain of Shigella) and ATCC 19112 (the strain of Listeria bacteria) obtained the best identification rate of 0.96. This study showed that Raman spectroscopy combined with machine learning could identify foodborne pathogens in foods by exploring the cells. Raman spectroscopy was also used for detecting other biological contaminants such as aflatoxin B1 in edible oils [54]. Deep learning algorithms (CNN and recurrent neural network (RNN)) were investigated to qualitatively and quantitatively detect aflatoxin B1 in the samples. Both CNN and RNN reached the recognition accuracy of 100% in identifying the aflatoxin B1 contamination degree of the edible oil, and RNN outperformed CNN for detecting aflatoxin B1 contamination level with an R_p_^2^ of 0.95 and an RPD of 4.86. In another study, Deng et al. [55] further detected aflatoxin B1 in maize based on Raman spectroscopy. A portable Raman spectroscopy system was applied to collect spectra information. Three methods (bootstrapping soft shrinkage (BOSS), variable combination population analysis (VCPA), and CARS) were investigated to identify valuable wavelengths. Then, SVM was established based on selected wavelengths for detecting aflatoxin B1 in maize. From the results, it can be found that the characteristic wavelengths performed better than full wavelengths. Finally, the CARS-SVM model obtained the best result with an R_p_^2^ of 0.9715 and an RPD of 5.8258. These studies demonstrated that Raman spectroscopy effectively detects biological contamination such as aflatoxin B1 in foods.

#### 3.4.2. Chemical Contamination

Raman spectroscopy has also been used to detect adulteration and additives in foods. For example, melamine, vanillin, and sugar were detected in non-dairy powdered creamer using Raman spectral imaging [6]. The spectra of the pure components at all concentrations and the corresponding contribution images were extracted via self-modeling mixture analysis (SMA), whereafter spectral information divergence (SID) values were used to identify the pure component spectra. These contribution images produced Raman chemical images, and binary images of the components at different concentrations were subsequently generated. Finally, the number of pixels in the binary images of each composition was highly correlated with the actual component concentrations, and the correlation coefficient was found to be 0.99 for all components. It indicated that Raman spectral imaging could effectively identify different components and predict the concentrations in a complex food matrix. In another study, a single-drop Raman imaging technique was proposed to semi-quantitatively detect multiple hazardous factors (melamine, sodium thiocyanate, and lincomycin hydrochloride) in a milk solution, with detection sensitivities of 0.1, 1, and 0.1 mg/kg, respectively [58]. The results of this study demonstrated the effectiveness of the Raman imaging technique in evaluating milk safety. Moreover, machine learning based on Raman spectroscopy was used to detect the adulteration of edible oils [56]. Nine machine learning algorithms (PCA-linear regression (PCA-LNR), L1 penalty-LNR, L2 penalty-LNR, elastic net penalty-LNR, PLS, PCA-RF, RF, PCA-boosting, and boosting) were established for the adulteration detection. The L2 penalty-LNR proved the best to detect adulterated edible oils with R^2^ of 0.984 for olive oil adulterated with soybean oil and 0.910 for avocado oil adulterated with canola oil. This study demonstrated the performance of Raman spectroscopy for the authentication and detection of contaminants in foods.

Furthermore, Raman hyperspectral imaging was investigated to detect three different chemical adulterants (benzoyl peroxide, alloxan monohydrate, and L-cysteine) in wheat flour [10]. Spectral angle mapping (SAM) was used to pre-process the data to distinguish adulterant pixels from the flour background. The visualization and detection of the adulterant pixels were carried out using binary images that had been converted from SAM images. The results showed that the proportions of adulterants in the wheat flour calculated using pixels corresponded to their added concentrations (correlation coefficients: 0.985, 0.985, and 0.987, respectively) and thereby demonstrated that Raman hyperspectral imaging is an accurate, effective, and non-destructive technique for detecting the authenticity of powdered foods.

#### 3.4.3. Physical Contamination

Fish bones, which can be hazardous to consumers, were also detected using Raman hyperspectral imaging by Song et al. [57]. The Raman spectra differences between fish bones and meat were investigated, and a fuzzy-rough set model based on the thermal-charge algorithm (FRSTCA) was established to select optimal wavebands. After that, support vector data description (SVDD) was established based on the selected wavebands (961 and 965 cm^−1^) to detect the fish bones, and the position and distribution of the fish bones in fish fillets were finally identified. The results showed that fish bones at a depth of less than 2.5 mm could be effectively detected, with a detection accuracy of 90.5%. It also provided a reference method for detecting other foreign bodies in foods.

## 4. Conclusions

This review discussed various spectral imaging techniques for detecting physical, chemical, and biological hazards in foodstuffs. Studies, such as those reviewed herein, have shown the use of Vis-NIR spectroscopy, THz spectroscopy, hyperspectral imaging, and Raman spectroscopy to detect both internal and external contamination of foods. It was shown that Vis-NIR spectroscopy usually performs well in detecting food biological and chemical contamination, THz spectroscopy performs well in detecting food chemical and physical contamination, and hyperspectral imaging and Raman spectroscopy work well for all types of food contamination. In addition, hyperspectral imaging can provide spatial imaging information, which is very useful for visualizing hazards in foodstuffs. Raman spectroscopy can obtain samples’ molecular structures and composition information and, thus, be utilized in microanalysis. Furthermore, it is effective in liquids since it is not affected by water within the samples. However, this technique is usually limited to very small sample volumes.

Consequently, using appropriate spectral imaging techniques is critical in detecting foodstuff hazards. Significantly, the advantages and disadvantages of spectral imaging techniques should also be considered. For example, the cost of a Vis-NIR sensor is less than that of a hyperspectral imaging sensor, and this technique is also easier for data collection and analysis. Thus, Vis-NIR outperforms hyperspectral imaging when only spectrum information is required. In the future, smaller, lighter sensors that are more sensitive to specific food contamination should be developed. For example, smartphone-based spectral imaging sensors could be helpful in the fast and portable detection of foodstuff hazards. Furthermore, more robust and reliable models based on machine learning algorithms should be studied for high-precision detection.

## Figures and Tables

**Table 1:** Features of spectral imaging techniques that are used in the detection of foodstuff hazards.

Techniques	Advantages	Disadvantages	References
UV-Vis-NIR spectroscopy	Easy operation, rapidity, non-destructivity, in situ, online, low cost, and portability	No imaging information and excessive noise	[15,16]
NIR spectroscopy	Easy operation, rapidity, non-destructivity, in situ, online, wide spectral range, and rich information on chemical bonds and groups	No imaging information	[15,17]
THz spectroscopy	Rapidity, reliability, non-destructivity, non-ionization, and with spectral fingerprinting characteristics	High cost, strong absorption of THz radiation due to water, scattering effects, limited penetration, limited sensitivity, and low LOD	[9,12]
Hyperspectral imaging	Rapidity, real time, non-destructivity, high spectral resolution, and a combination of spectral and imaging information	Complicated image analysis and redundant information	[8,16,18]
Raman spectroscopy	High specificity, high sensitivity, simplicity, non-sensitivity to water, and evident Raman fingerprint of target attributes	Limited to very small sample volumes and inability to acquire information from large surface areas	[6,16,19]

**Table 2:** Applications of spectral imaging-based techniques for foodstuff hazards detection.

Foods	Techniques	Hazards	Models and Algorithms	Results	References
Corn kernels	UV-Vis-NIR spectroscopy	Aflatoxin	RF	Training: 95.3%; Testing: 94.8%	[21]
Wheat kernels	Vis-NIR spectroscopy	Toxigenic fungi	PCA, LDA, and PLS	Different fungal strains: 75–100%, Different infection levels: 88.3–100%, R_p_^2^ = 0.89	[22]
Peanut kernels	Vis-NIR spectroscopy	Aflatoxin B1	SNV, random frog, and PLS-DA	Full wavelengths: 88.57% and 92.86%; Selected wavelengths: 90% and 94.29%	[23]
Corn	FT-NIR spectroscopy	Aflatoxin B1	ACO, NSGA-II, and BPNN	The best correlation coefficient in prediction: 0.9951	[24]
Hami melon	Vis-NIR spectroscopy	Pesticide residues (chlorothalonil, imidacloprid, and pyraclostrobin)	1D CNN, CNN, PLS-DA, and SVM	Identification accuracies of 1D CNN in test sets: 95.83% for four-class; 99.17% for two-class	[25]
Rough, brown, and milled rice	NIR spectroscopy	Pesticide residues (chlorpyrifos-methyl)	PLS, mean centering, SNV, MSC, and derivative	Quantitative detection: R^2^ = 0.702–0.839 for rough rice, 0.722–0.800 for brown rice, and 0.693–0.789 for rough rice; Qualitative detection: correct classification = 77.8–92.6% for rough rice, 79.6–88.9% for brown rice, and 94.4–100% for milled rice	[26]
Strawberries	NIR spectroscopy	Pesticide residues (boscalid and pyraclostrobin)	PLS, PCA, 1st and 2nd derivative, MSC, and SNV	Correlation coefficients: 0.93 for boscalid and 0.83 for pyraclostrobin	[27]
Chinese kale, cabbage, and chili spur pepper	NIR and MIR spectroscopy	Pesticide residues (profenofos)	PLS, 1st derivative, and SNV	R^2^ = 0.97 for Chinese kale, 0.88 for cabbage, and 0.96 for chili spur pepper	[28]
Honey	NIR spectroscopy	5-HMF	MSC, SNV, 1st and 2nd derivative, Savitzky-Golay smoothing, PCR, and PLS	The best result: R_p_^2^ = 0.98 and RPD = 3.3	[29]
Potatoes	Vis-NIR spectroscopy	Acrylamide	SNV, MSC, Savitzky-Golay filtering, 1st and 2nd derivative, feature standardization, sequential forward selection (SFS), NB, LDA, SVM, KNN, PLS, RF, quadratic discriminant analysis (QDA), extreme learning machine (ELM), decision tree (DT), boosted tree (BT), and neural network (NN)	Classification of LDA: 92%	[30]
Bok choi	NIR spectroscopy	Chlorpyrifos	Savitzky-Golay smoothing, mean normalized, SNV, baseline correction, MSC, 1st derivative, 2nd derivative, PLS-DA, SVM, ANN, and PC-ANN	The best accuracy, precision, recall, and F1-scores: 1.0	[31]
Rice powder	THz	Pesticide residue (carbendazim)	SVM, PLS, and SVR	Qualitative detection: 100%; Quantitative detection: R = 0.9978	[32]
Wheat flour	THz	Benzoic acid	GRNN, BPNN, and PCA	Correlation coefficient: 0.85	[33]
Milk powder	THz	Melamine	PLS and MLR	R^2^ = 0.98 for PLS and 0.97 for MLR	[34]
Wheat flour	THz	Pesticide residues (6-Benzylaminopurine, 2,6-Dichlorobenzonitrile, and imidacloprid)	BPNN, SVR, GA, and PSO	The best correlation coefficients: 0.9913, 0.9948, and 0.9923	[35]
Sausages	THz	Foreign materials (aluminum shards)	PCA and DA	98.3–100%	[36]
Wheat grain	THz	Foreign bodies (a stone, a metal screw, a glass fragment, and a wood chip)	Linear low-pass filtering and non-linear anisotropic diffusion	/	[37]
Sugar and milk powder	THz	Foreign substances (insects)	/	/	[38]
Zizania latifolia, rice, and maize	THz	Pesticide residues (2,4-dichloro phenoxy acetic acid)	AsLS, AirPLS, Backcor, and BEADS	/	[39]
Walnuts	THz	Endogenous foreign bodies (shells)	PCA	Classification: 95%	[9]
Chocolate bars, dried laver, red ginseng, and walnuts	THz	Foreign bodies (metal washer, rubber band, pepper seed, and polystyrene pieces)	/	Well discriminated	[40]
Fish	THz	Endogenous foreign bodies (fish bones) and exogenous foreign substances (metal, plastic, and wooden toothpicks)	CARS, UVE, SPA, PLS-DA, LDA, and SVM	The best detection result: 99.56%	[41]
Pistachio kernels	Hyperspectral imaging	Aflatoxin B 1	SNV, Savitzky-Golay smoothing, PCA, LDA, and SMLR	Classification: 92.5% (calibration) and 91% (validation); Prediction: higher than 0.91 (calibration and validation)	[42]
Wheat kernels	Hyperspectral imaging	Deoxynivalenol	PLS, LDA, PCA, and SNV	Full cross-validation: R_cv_^2^ = 0.72; Independent validation: R_p_^2^ = 0.27; Correct classification accuracy: 62.7%	[11]
Maize kernels	Hyperspectral imaging	Aflatoxin B1	PLS-DA, KNN, PCA, PLS, MSC, SNV, and Savitzky-Golay smoothing	Classification: 98.2%; Prediction: R_cv_^2^ = 0.82	[43]
Peanut kernels	Hyperspectral imaging	Aflatoxin B1	1D modified TCN, 1D TCN, 1D LSTM, and 1D CNN	The best accuracies: 99.60% in training and 99.26% in testing by 1D modified TCN	[44]
Lettuce	Hyperspectral imaging	Pesticide residues (fenvalerate and dimethoate)	LS-SVR, CARS, RF-RFE, SPA, and SNV	CARS-SPA-LS-SVR (fenvalerate): R_p_^2^ = 0.8890; RF-RFE-SPA-LS-SVR (dimethoate): R_p_^2^ = 0.9386	[45]
Fresh-cut potato slices	Hyperspectral imaging	Sulfur dioxide residue	SVM, PCA, 2nd derivative, and Savitzky-Golay smoothing	Full wavelengths: 98.75% in calibration and 95% in prediction; Selected wavelengths: 99.38% in calibration and 92.50% in prediction	[46]
Garlic chive	Hyperspectral imaging	Pesticide residues (λ-cyhalothrin, trichlorfon, and phoxim)	1D CNN, KNN, LDA, NB, RF, and SVM	1D CNN: 98.5% in training and 97.9% in testing	[47]
Beef	Hyperspectral imaging	Veterinary drug residues (metronidazole, ofloxacin, salbutamol, and dexamethasone)	CNN, MLP, SVM, RF, CARS, PCA, and DWT	Overall accuracies: 91.6%, 88.6%, 87.6%, and 86.2%	[48]
Chicken meat	Hyperspectral imaging	Bone fragments	PCA	Detection accuracy: 93.3%	[8]
Seaweed	Hyperspectral imaging	Insect, shrimp shell, thread, feather, and plastic bag	The proposed algorithm and SVM	The proposed algorithm: 95%; SVM: 79%	[49]
Broiler breast meat	Hyperspectral imaging	Foreign materials (polymer, wood, and metal)	Fusion model, PCA, Savitzky-Golay smoothing, Gap Segment 2nd derivative, and SNV	Classification accuracies of 2 × 2 mm^2^: 95%, 95%, and 81%; Classification accuracies of 5 × 5 mm^2^: 100%, 100%, and 100%	[50]
Chinese hickory nuts	Hyperspectral imaging	Endogenous foreign bodies (shell fragments)	2D CNN-LSTM, KNN, SVM, and PCA	2D CNN-LSTM obtained the best overall classification accuracy of 99%.	[51]
Soy protein meat	Hyperspectral imaging	Foreign bodies (polylactic acid, polypropylene, polyethylene terephthalate, and polyvinyl chloride)	SNV, Savitzky-Golay smoothing, 1st derivative, 2nd derivative, MSC, PCA, SPA, CARS, LDA, KNN, BP-ANN, and SVM	MSC-PCA-SPA-SVM obtained the best classification accuracy: 95.00%	[52]
/	Raman spectroscopy	Foodborne pathogens (Escherichia, Listeria, Vibrio, Shigella, and Salmonella)	GA, PSO, and ANN	The average accuracies: 0.89 (GA-ANN) and 0.93 (PSO-ANN); The best identification rate: 0.96	[53]
Edible oils	Raman spectroscopy	Aflatoxin B1	CNN and RNN	Qualitative detection: 100%; Quantitative detection: R_p_^2^ = 0.95 and RPD = 4.86	[54]
Maize	Raman spectroscopy	Aflatoxin B1	BOSS, VCPA, CARS, and SVM	R_p_^2^ = 0.9715 and RPD = 5.8258	[55]
Edible oils	Raman spectroscopy	Adulterated oils	PCA-linear regression (PCA-LNR), L1 penalty-LNR, L2 penalty-LNR, elastic net penalty-LNR, PLS, PCA-RF, RF, PCA-boosting, and boosting	R^2^ = 0.984 for olive oil adulterated with soybean oil and 0.910 for avocado oil adulterated with canola oil	[56]
Wheat flour	Raman hyperspectral imaging	Benzoyl peroxide, alloxan monohydrate, and L-cysteine	SAM, ICA, and Kruskal-Wallis test	Correlation coefficients: 0.985, 0.985, and 0.987	[10]
Fish fillets	Raman hyperspectral imaging	Fish bones	FRSTCA and SVDD	Classification accuracy: 90.5%	[57]
Non-dairy powdered creamer	Raman spectral imaging	Melamine	SMA and SID	Correlation coefficient: 0.99	[6]
Milk solution	Raman imaging	Melamine, sodium thiocyanate, and lincomycin hydrochloride	DWT	Detection sensitivities: 0.1, 1, and 0.1 mg/kg	[58]

## Data Availability

The data presented in this study are available on request from the corresponding author.
